# Cross-National Interactions Between Absolute and Relative Education Across Sex/Gender

**DOI:** 10.1093/geroni/igaf122.1513

**Published:** 2025-12-31

**Authors:** Mateo Farina, Chloe Eng, Arthur Wang, Laura Zahodne, Usha Dhakal, Justina Avila-Rieger, Lindsay Kobayashi, Jennifer Manly

**Affiliations:** University of Texas at Austin, Austin, Texas, United States; Stanford University, Stanford, California, United States; University of Southern California, Los Angeles, California, United States; University of Michigan, Ann Arbor, Michigan, United States; Georgetown University, Washington, District of Columbia, United States; Columbia University Irving Medical Center, New York, New York, United States; University of Michigan, Ann Arbor, Michigan, United States; Columbia University Irving Medical Center, New York, New York, United States

## Abstract

The global burden of dementia is expected to grow in the coming decades, impacting the lives of millions. However, the determinants of dementia and the strengths of their associations are not universal but likely vary across country contexts. Understanding this variability in key determinants of cognitive health like education is paramount for understanding the population dynamics of this growing public health crisis. Specifically, the association between education and cognitive health can vary due to distribution, quality, and opportunities within countries. Using data from the Harmonized Cognitive Assessment Protocol, we examine variability in the association between absolute and relative levels of education, and their interaction, for older men and women in the United States, United Kingdom, India, Mexico, and South Africa. We use Multilevel Analysis of Individual Heterogeneity and Discriminatory Accuracy (MAIHDA), which allows for a complex modeling approach that accounts for additive and multiplicative interactions across country context. We examine these models across global cognitive functioning as well as specific domains (memory, language, executive functioning). Preliminary findings show strong main effects of relative and absolute levels of education on global cognitive functioning and across domains. We also find that strong evidence of interactive effects across country context. For example, women with high relative education and high absolute levels overperformed in Mexico, while women with high relative education regardless of absolute levels underperformed in South Africa. Overall, these findings point to the importance of considering country context (i.e. educational quality, socioeconomic opportunities, and larger policy environment) when examining determinants of dementia.

